# Prevalence and factors associated with disruptive behavior among Iranian students during 2015: a cross-sectional study

**DOI:** 10.1186/s13052-020-00848-x

**Published:** 2020-06-18

**Authors:** Marzieh Araban, Ali Montazeri, L. A. R. Stein, Mahmood Karimy, Ali Asghar Haeri Mehrizi

**Affiliations:** 1 Department of Health Education and Promotion, Social Determinants of Health Research Center, Public Health School, Ahvaz Jundishapur University of Medical Sciences, Ahvaz, Iran; 2 Health Metrics Research Center, Iranian Institute for Health Sciences Research, ACECR, Tehran, Iran; 3 Psychology Department, University of RI, Kingston, RI USA; 4 Behavioral & Social Sciences Department, Brown University School of Public Health, Providence, RI USA; 5 RI Training School, Cranston, RI USA; 6 Social Determinants of Health Research Center, Saveh University of Medical Sciences, Saveh, Iran

**Keywords:** Youth, Prevalence, Disruptive behavior, Perceived social support, Perceived vulnerability, The Snyder hopes scale

## Abstract

**Background:**

Disruptive behavior can have lifetime consequences for youth. Prevention, early identification and treatment of disruptive behavior can improve outcomes for these youth. The purpose of the present study was to assess the prevalence of disruptive behavior among a sample of Iranian youth, and the relationship of disruptive behavior to other psychological phenomena that may be targeted for prevention, early identification and treatment.

**Method:**

The sample consisted of 600 high school students (300 boys and 300 girls; ages 15 to 18 years old) selected through multi-stage random sampling in Saveh city, of Iran, in 2015. Questionnaires assessed several phenomena including demographics, life satisfaction, social support, depression, stress, smoking and hopefulness. The Disruptive Behavior Scale was also utilized. Univariate analyses were followed by multiple logistic regressions to examine relations among disruptive behavior and other constructs.

**Results:**

Prevalence of disruptive behavior was 7.5%, in boys and 3.1%, in girls. Mean scores were 22.97 ± 1.17 for boys and 19.15 ± 1.06 for girls, with a significant difference between them (*P* < 0.05). The results of regression revealed low life satisfaction (OR = 3.75; 95% CI: (2.37–5.91), social support (OR = 0.72; 95% CI: (0.56–0.82) and hopefulness (OR = 0.85; 95% CI: (0.62–0.92); and smoking (OR = 3.65; 95% CI: (2.19–6.06), being male (OR = 2.55; 95% CI: (1.54–4.22), and higher stress (OR = 1.92; 95% CI: (1.60–2.91) and depression (OR = 2.76; 95% CI: (1.82–4.88) were significant factors in predicting disruptive behavior.

**Conclusion:**

Disruptive behavior was associted with life satisfaction, smoking, being a boy, social support, hopefulness, stress, and depression. Targeting constructs (e.g., support, stress) associated with disruptive behavior may assist in prevention, early identification and treatment of problem behavior. For example, health promotion programs to increase hopefulness, satisfaction and support, and reduce stress, depression and smoking might be of importance for prevention and treatment of disruptive behavior.

## Introduction

Disruptive behavior disorders (DBDs) is defined as “student behavior that systematically disrupts educational activities, undermines the habitual development of the tasks carried out in the classroom and causes teacher to invest a significant amount of time in dealing with it, time that should otherwise be devoted to the processes of teaching and learning” [1]. DBD’s definition based on DSM-5 is a repetitive and persistent pattern of behavior in which the basic rights of others or major age-appropriate societal norms or rules are violated, as manifested by the presence of criteria such as aggression to people or animals, and destruction of property [2]. Disruptive behavior has different forms. One example is the student who talks continually while the teacher is teaching, interrupts the class by asking questions and making different sounds, uses different forbidden gadgets like cell phones in class [3] and becomes angry when the teacher opposes his/her inappropriate behavior [4].

Early onset DBD can have life-time consequences, including school absences, poor school achievement, substance use, aggression and anxiety; and DBD tends to continue to adulthood [5]. Adolescents with DBD have low self-control, conflictual relationships, and low empathy. These youth have difficulty with interpersonal relationships, and managing behavior, putting them at high risk for violence and substance abuse [6].

A 2016 survey in Amsterdam revealed that the most prevalent disorders among adolescents were disruptive behaviors [7]. Prevalence rates were 8.5% according to the DSM-IV and 7.1% according to the ICD-10 in Brazilian youth in 2010 [8]. Most studies in this field are from western countries. For example, a 2012 Dutch population study indicated a mean prevalence rate of 12.8% for DBDs; with 9.3% for girls and 15.2% for boys [9]. Although a 2016 community-based study in Iranian children and adolescents revealed the prevalence of psychiatric disorders was 10.55%, the study did not specifically screen for disruptive behavior and did not attend to gender differences in prevalence rates. In addition, this study did not include youth attending schools in non-capital cities, nor did it include important psychosocial factors [10] that might be targeted for prevention, early detection or treatment. Despite problems resulting from disruptive behavior, it has received little attention in the literature [11]. Furthermore, compared to boys, the study of contributing factors of disruptive behavior in girls is under developed [1]. As such, it is important to identify possible predictors of disruptive behavior in both boys and girls in order to establish prevention and treatment programs [12].

It has been reported that almost 22% of children and adolescents suffer from some form of psychiatric disorder [13]. In a 1997 study of Iranian elementary school children, %1.8% of boys and 12.1 of girls had disruptive behavioral disorders [14]. In another study in Iranian Children and Adolescents in 2016, the prevalence of oppositional defiant disorder (ODD) was 4.45 [13]. There is a strong need to better understand the prevalence of mental disorders, and to understand factors related to mental disorders, in children and adolescents in Iran. Addressing mental health services needs is a priority. Understanding psychiatric disorders in the context in which they occur is necessary in order to provide effective psychiatric services [15]. Although many studies have been carried out on disruptive behavior in western countries, no study has investigated the prevalence of disruptive behavior using a culturally adapted instrument so far in Iran. Additionally, studying psycho-social phenomena associated with DBD may assist in better understanding how to mitigate this behavior disorder [16]. For example one study showed depressive symptoms mediated the relation between marijuana use and disruptive behavior [17], whereas another found that personal characteristics, such as maladaptive parenting, predict disruptive behavior [18]. Therefore, the purpose of the present research was to evaluate the prevalence of disruptive behavior, and its association with other psychological phenomena in a sample of Iranian youth.

## Methods

### Study design

This was a cross-sectional study. The research was conducted in Saveh city, center of Iran. The adolescent population is estimated at 47425 inhabitants. Of students invited to participate, the response rate was 98% (600 out of 612 surveyed among 10-12th grade students). Students completed paper-and-pencil, self-administered questionnaires in their classrooms. Questionnaires were delivered in a packet, with the same unique identification number on each questionnaire within the packet. Student identifying information was not collected. Questionnaires were completed in the presence of a researcher who explained the procedures and the aim of study. Teachers left the schoolroom during completion of questionnaires. Students took 20 min to answer the questions. Once completed, students put their questionnaires into a box in order to maintain anonymity.

### Participants and sampling

The study consisted of 600 students —from 15 to 18 years old, who lived in Saveh, Iran in 2015. There were 300 female participants and 300 male participants in the research. Approximately 83% (*n* = 503) of adolescents were born in the Saveh City, whereas all other adolescents were born outside of the Saveh City. Written informed consent was obtained from parents (youth provided assent). All procedures were reviewed and approved by both the Saveh University of Medical Science and the Ministry of Education)Saveh county department of education). The research team clarified to participants that their answers would remain confidential. Participant inclusion criteria were: Ability to provide informed assent/consent, aged 15–18 years and, attending high school in Saveh city. There are no specific exclusion criteria, other than that the participants must be willing to participate and comply with the study protocol.

The sample was obtained using multistage sampling with three stages. Multistage sampling methods can be used to recruit participants in experimental or observational studies. Schools were selected from 32 high schools from two city regions. Each school was given a specific number. Using a random numbers table, 4 high schools (2 girl high schools and 2 boy high schools) were selected from each region, which constituted a total of 8 high schools. The quota of students from any school was based on the proportion of the students in the school, and then all classes were included in the selected schools. In addition, from each school, equal numbers of the student in each grade were selected. Finally, subjects were selected randomly from each class based on their identification numeral.

### Measures

1. **Demgraphics questionnaire**: This questionnaire contained 15 items on age, gender, smoking status (yes/no), housing status, scores at school, number of friends, pocket money, parents’ jobs, parents’ education levels, and life satisfaction)yes/no). 2. **Disruptive Behavior Scale for Adolescents (DBSA)**: This questionnaire was comprised of four constructs derived from 29 items [19, 20]. Reponse options were rated on a four-point scale ranging from 0 (never) to 3 (always). The four constructs with sample items include: Intentional Violations – “I deliberately break or damage school equipment;” Mistakes – “I make noise and disrupt the class;” Distraction/Transgression – “I don’t turn up on time for school;” and Aggression to School Authorities – “I argue with school authorities.” Higher scores indicate higher level of disruptive behaviors. The reliability of the instrument was confirmed using Cronbach alpha coefficient (Intentional Violations = 0.82, Mistakes = 0.91, Distraction/Transgression = 0.77, Aggression to School Authorities = 0.86). Validity of this questionnaire was demonstrated through content and construct validity. Content Validity Ratio and Content Validity Index were confirmed with 0.82 and 0.87 respectively. The model’s fit was confirmed for all scales (goodness-offit index > 0.90) [21]. 3. **Perceived social support**: This was assessed using the 12-item instrument (sample item, “Every time I^,^ve needed it, I^,^ve always found a certain person to be there for me”) developed by Zimet et al. [22]. Response options range from 0 (very strongly disagree) to 6 (very strongly agree). Reliability of the Farsi version of the instrument has been found to be 0.84 for the scale [23]. In the present study, Cronbach’s alpha scores for the scale have obtained the level of 0.84. 4. **Perceived vulnerability**: This measure is composed two scales [24] including perceived depression (4 items) and stress (3 items). Reponse options range from 0 (never) to 3 (always). In this study scales showed good internal consistency with Cronbach’s alpha of 0.89. Cronbach’s alpha coefficients, in the previous study in Iran, were found to be 0.79 [25]. 5**. The Snyder Hope Scale:** This scale includes 8 items (sample itme, “I usually find myself worrying about something”) rated from Definitely False (1) to Definitely True (8). This scale is valid for use in Iran, and reliability of the Farsi version of the instrument has been found to be 0.82 [26]. In our study reliability was confirmed through a Cronbach’s alpha value of 0.78.

### Statistical analysis

Data were analyzed with Statistical Package for Social Sciences-version 15 (SPSS-15) software, International Business Machines Corporation (IBM) located in the United States. Before analysis, data were examined using histograms, the Kolmogorov-Sminov test, and normality of residuals. All were normaly distributed. Demographic data were subjected to simple descriptive analyses. One-way analysis of variance (ANOVA) and independent sample t-tests were performed to examine significant differences between DBD mean scores by gender, education level, and so forth. Correlations were performed between continuous variables to determin associations with DBD (e.g., hopefulness and DBD). Multiple logistic regression was used to determine constructs that were signficantly associated with DBD. In order to identify the effects of social support, hopefulness, perceived stress and depression and demographic variables (e.g. education, gender, etc) a multiple unconditional logistic regression analysis was conducted, with disruptive behavior as the dependent variable. In the multiple logistic regression model, only variables significantly associated with disruptive behavior in univariate analysis were included (e.g., gender, smoking, life satisfaction, social support, hopefulness, perceived depression and stress, scores at school and parent education). To conduct logistic regression, we coded scores less than the mean as 0, and scores more than or at the mean as 1 [27]. Logistic regression is a widely used test to assess independent effects of a variable on binomial outcomes in medical literature [28, 29]. *P*-values less than or equal to 0.05 were considered significant.

### Ethics

All participants were informed about study confidentiality. Informed consent was obtained from all the participants and/or their parents. The study was approved by the ethics committee of Saveh University of Medical Sciences.

## Results

### Sample description

Participants consisted of 600 adolescents aged 15 to 18 with a mean age of 16.7 ± 0.87 years, with equal numbers of males and females. Of students, 16.7% were in the last year of high school (seniors) and 40 and 43.3% were in first and second year of high school (freshman and juniors) respectively. It should be noted that in Iran we have 3 grade levels (10th, 11th, 12th grades; or freshman, junior, senior, respectively). Regarding the housing status, 91.8% of students were living with parents, 5.2% with one parent, and the rest with the grandfather or grandmother or others. More than half of the students (58%) reported feeling “life satisfaction” in the past 12 months. Prevalence of smoking experience was 26% (Table 1).

**Table 1:** Characteristics of Study Sample

Variable	N	%	Mean	Standard Deviation
Gender				
Female	300	50.0	19.1	1
Male	300	50.0	22.9	1.1
Number of friends				–
1–2	143	23.8	20.4	2.1
3–4	297	49.5	20.9	1.6
≥ 5	160	26.7	21.3	2.0
Smoking				
Yes	155	26.0	24.1	1.2
No	445	74.0	18.4	2.2
Life satisfaction				
Yes	348	58.0	19.0	2.3
No	252	42.0	23.2	1.9
Education				
First high school	240	40.0	22.1	1.2
Second year	260	43.3	21.8	2.1
Last year	100	16.7	22.0	1.7
Average school score				
< 14	206	34	23.1	2.2
> 15	394	56	20.0	1.4
Pocket money				
≤ 10000Rial	62	10.3	21.7	1.7
10-30000Rial	159	26.5	21.6	1.4
30-50000Rial	178	29.6	20.3	1.9
≥ 50000Rial	201	33.5	22.3	2.2
Housing status				
With parent	551	91.8	20.2	2.9
One parent	31	5.2	20.6	2.6
Grandparents	6	1.0	21.2	2.1
Others	12	2.0	21.3	2.7
Mother job				
Household	507	84.5	21.0	1.8
Employed	93	15.5	20.5	2.0
Father job				
Employee	96	16.0	20.0	2.0
Worker	252	42.0	21.5	2.0
Retired	26	4.3	20.9	1.5
Self-employment	226	37.7	21.1	1.6
Father’s education				
University	65	11.0	23.9	2.1
Secondary school	382	63.7	21.4	2.3
Elementary/ illiterate	153	25.5	19.2	2.4
Mother’s education				
University	77	12.8	24.3	2.2
Secondary school	279	36.5	22.6	2.1
Elementary/ illiterate	244	40.7	20.8	2
Disruptive behavior	–	–	21.0	1.1
Social support	–	–	38.8	8.4
Perceived stress	–	–	5.0	2.3
Perceived depression	–	–	5.2	2.6
Hopefulness	–	–	45.7	7.8

The prevalence of disruptive behavior was 7.5%, in boys and 3.1%, in girls; also, average score of disruptive behavior for all participants was 21.17 ± 1.94. This score was 22.97 ± 1.17 for boys and 19.15 ± 1.06 for girls, with a significant difference between them (*P* < 0.05). Means and standard deviations of subscales of disruptive behavior including Intentional violations, Distration/transgression, Mistakes, and Aggression to school authorities was 8.5 ± 8.1, 4.6 ± 4.2, 5.2 ± 5.5 and 3.0 ± 4.1 respectively. Significant differences were not found among the scores of boys and girls in constructs (subscales) of disruptive behavior except the intentional violations construct. The mean score of disruptive behavior was significantly higher for smokers than non-smokers; and independent sample t-tests showed that there were significant differences between non-smokers and smokers in all constructs of disruptive behavior. Disruptive behavior mean score was significantly higher for youth with less life satisfaction that for those with more life satisfaction; and similarly, disruptive behavior score was significantly higher for students with lower school scores than for students with higher school scores. Finally, mean disruptive behavior score differed significantly by mother (and separately by father) education using analysis of variance. Disruptive behavior scores were not associated with living situation, number of friends, pocket money or parent employment (Table 1). Univariate tests indicated a significant relationship between each of the following constructs and DBD using correlations (*P* ≤ .05): Social support, hopefulness, stress and depression.

Only variables significantly associated with disruptive behavior (*P* < 0.05), including: Gender, parent education, school scores, smoking status, life satisfaction, social support, hopefulness, perceived stress and perceived depression were entered in furthur analysis. In multiple logistic regression analysis, results of the Hosmer and Lemeshow test showed acceptable goodness of fit of the model (*P* > 0.05). Results of multiple unconditional forward logistic regression analysis revealed that the following constructs were significantly associated with disruptive behavior: Being male (odd ratio [OR] = 2.55; 95% confidence interval [CI]: (1.54–4.22), smoking (OR = 3.65; 95% CI: (2.19–6.06), lower life satisfaction (OR = 3.75; 95% CI: (2.37–5.91), social support (OR = 0.72; 95% CI: (0.56–0.82) and hopefulness (OR = 0.85; 95% CI: (0.62–0.92); and more perceived stress (OR = 1.92; 95% CI: (1.60–2.91) and depression (OR = 2.76; 95% CI: (1.82–4.88). See Table 2.

**Table 2:** Results of the multiple logistic regression analysis

	B	S.E	Wald	OR	95% CI	P
**Demographics**						
Gender						
girl					1.0 (Ref.)	
boy	0.939	0.256	13.47	2.55	1.54–4.22	0.001
Smoking						
no					1.0 (Ref.)	
yes	1.295	0.259	24.99	3.65	2.19–6.06	0.001
Life satisfaction						
yes					1.0 (Ref.)	
no	1.322	0.232	32.44	3.75	2.37–5.91	0.001
Social support	0.326	0.527	0.367	0.72	0.56–0.87	0.01
Hopefulness	0.156	0.146	1.139	0.85	0.63–0.92	0.001
Perceived stress	0.668	0.289	5.451	1.92	1.60–2.91	0.001
Perceived depression	1.022	0.360	8.01	2.76	1.82–4.88	0.005

## Discussion

This study aimed to determine the prevalence of disruptive behavior among a sample of Iranian youth and the relationship of disruptive behavior to other psychological phenomena. Identifying factors associated with disruptive behavior in classrooms can be helpful in improving community health [3]. According to results of this study, significant gender differences in disruptive behavior among Iranian adolescents were revealed, which is consistent with previous research in other countries on adolescent disruptive behavior [30, 31]. This result may be due to the relatively higher levels of parental monitoring of girls as compared to boys in Iranian culture. This finding may also be related to relatively higher testosterone levels found in male as compared to female adolescents, as testosterone has been linked to aggression [32].

Similar to previous studies, results of this study demonstrate that life satisfaction is negatively related with adolescent problem behaviour [33–35], and that perceived stress and depression levels are positively associated with disruptive behavior. For example, Estevez et al. showed that aggressive behavior in adolescence has been significantly related to high levels of perceived stress, depressive symptoms and low life satisfaction [36]. In a study by Musitu et al. perceived stress was significantly associated with student aggression [37]. Another study by Desousa et al. showed that life satisfaction was negatively related with adolescent problem behavior [35]. In addition, McKnight et al. demonstrated that life satisfaction mediated the association between stressful life events and adolescent problem behaviour [38]. In another study, Suldo and Huebner found that life satisfaction had a mediating effect between adolescent problem behavior and parental involvement [39].

In our study, there were significant differences between smokers and non-smokers in disruptive behavior, with smokers having higher mean scores. Results of logistic regression analysis indicated smoking was significantly associated with disruptive behavior. Similar results have been reported in previous researchs [34, 40]. For instance, in study of Upadhyaya et al., high rates of disruptive behavior disorders were found in adolescent smokers [40].

Social support has been related with positive mental health outcomes in many populations, including adolescents with disruptive behavior. Social support provided by important others affects an individual’s actual and perceived behavioral control [41]. Consistent with other research, our study indicated that increased preceived social support decreased likelihood of disruptive behavior. Similarly, Forouzan, et al. found that social support promotes healthy behaviors in an individual’s life [42], including prosocial behaviors that are inconsistent with disruptive behaviors. Results of our study indicated that youth hopefulness is also associated with disruptive behavior. Hope has been found to be an important factor in good behavioral and mental health [26]. Adolescents with high levels of hope evidence better general health maintenance, problem solving, and mental health [43].

To summarize, results of univariate tests demonstrated the following factors were associated with higher levels of disruptive behavior: Being male; smoking; less life satisfaction, hope and social support; and higher stress and depression. This is consistent with prior research outside of Iran, and it is important to demonstrate similar associations within Iran so that existing interventions might be adapted to Iranian culture. Of note, when these factors were enterred into multiple logistic regression, parent education and grades were no longer significant. Results of logistic regression indicate that life disatisfaction, smoking and depressive symptoms were among the constructs most highly associated with disruptive behaviors.

Given the design of the study, we cannot say whether disruptive behavior causes these associated problems (e.g., depression, less social support), whether these problems cause disruptive behavior, or whether some third factor causes a cluster of poor behaviors (e.g., poor parential monitoring contributes to later smoking and disruptive behavior). However, this study suggests that interventions to improve disruptive behavior in youth may also benefit by first targeting and improving life satisfaction, reducing smoking as appropriate, and treating depressive symptoms. More longitudianl work is needed to establish causal effects among these constructs.

### Limitations

There are several limitations of the current study. Participants were recruited from high school. Thus, findings may not extend to the general adolescent population, or to youth with severe disruptive behavior who may not attend school. On the other hand, it may behoove researchers and clinicians to study disruptive behavior in youth not yet severely disordered, and in settings like schools where problem behavior can have consequences for an entire class. Secondly, results rely on self-report, so that youth may under or over-report behaviors, although we believe this is somewhat mitigated with assurances of anonymity. Third, although number of friends was not associated with disruptive behavior, it may be that type of friend (i.e., delinquent vs prosocial friend) is. Fourth, living situation was also not found to be associated with disruptive behaviors, but it may be that there was not enough variability in the sample (e.g., over 90% lived with both parents). Finally, data were cross-sectional, therefore as stated above, causal associations cannot be inferred.

## Conclusions

Disruptive behavior in high school students is comparable to rates found in prior studies; and social support, hopefulness, stress, depression, gender, smoking and life satisfaction were significantly associated with disruptive behavior. Results may be of interest to the Ministry of Health, and the Ministry of Education and Training in terms of demonstrating the prevalence of disruptive behaviors in boys and girls, and identifying and adapting interventions that address disruptive behaviors and associated constructs (i.e., smoking, depression, life satisfaction). Health promotion programs might be of importance for prevention and treatment of disruptive behavior. Conducting longitudinal studies are recommended to better understand causal relations among disruptive behavior and different psychosocial variables in adolescents.

## Data Availability

Upon request, we can offer onsite access to a part of data analyzed at Saveh University of Medical Sciences, Saveh, Iran. To do so, Dr. Karimy should be contacted.

## Abbreviations

CVI: Content validity index
CVR: Content validity ratio
